# Evaluation and validation of a robust single cell RNA-amplification protocol through transcriptional profiling of enriched lung cancer initiating cells

**DOI:** 10.1186/1471-2164-15-1129

**Published:** 2014-12-17

**Authors:** Dominic G Rothwell, Yaoyong Li, Mahmood Ayub, Catriona Tate, Gillian Newton, Yvonne Hey, Louise Carter, Suzanne Faulkner, Massimo Moro, Stuart Pepper, Crispin Miller, Fiona Blackhall, Giulia Bertolini, Luca Roz, Caroline Dive, Ged Brady

**Affiliations:** Nucleic Acid Biomarker Laboratory, Clinical & Experimental Pharmacology, CR-UK Manchester Institute, University of Manchester, Manchester, M20 4BX UK; Computational Biology Support, CR-UK Manchester Institute, University of Manchester, Manchester, M20 4BX UK; Molecular Biology Core Facility, CR-UK Manchester Institute, University of Manchester, Manchester, M20 4BX UK; Department of Experimental Oncology, Tumor Genomics Unit, Fondazione IRCCS Istituto Nazionale dei Tumori, Milano, 20133 Italy; RNA Biology Group, CR-UK Manchester Institute, University of Manchester, Manchester, M20 4BX UK; Christie NHS Foundation Trust, Institute of Cancer Sciences, University of Manchester, Manchester, M20 4BX UK

**Keywords:** RNA-Amplification, Single cell, Transcriptional profiling, RNA-Seq, Microarray, Cancer initiating cell

## Abstract

**Background:**

Although profiling of RNA in single cells has broadened our understanding of development, cancer biology and mechanisms of disease dissemination, it requires the development of reliable and flexible methods. Here we demonstrate that the EpiStem RNA-Amp™ methodology reproducibly generates microgram amounts of cDNA suitable for RNA-Seq, RT-qPCR arrays and Microarray analysis.

**Results:**

Initial experiments compared amplified cDNA generated by three commercial RNA-Amplification protocols (Miltenyi μMACS™ SuperAmp™, NuGEN Ovation® One-Direct System and EpiStem RNA-Amp™) applied to single cell equivalent levels of RNA (25–50 pg) using Affymetrix arrays. The EpiStem RNA-Amp™ kit exhibited the highest sensitivity and was therefore chosen for further testing. A comparison of Affymetrix array data from RNA-Amp™ cDNA generated from single MCF7 and MCF10A cells to reference controls of unamplified cDNA revealed a high degree of concordance. To assess the flexibility of the amplification system single cell RNA-Amp™ cDNA was also analysed using RNA-Seq and high-density qPCR, and showed strong cross-platform correlations. To exemplify the approach we used the system to analyse RNA profiles of small populations of rare cancer initiating cells (CICs) derived from a NSCLC patient-derived xenograft. RNA-Seq analysis was able to identify transcriptional differences in distinct subsets of CIC, with one group potentially enriched for metastasis formation. Pathway analysis revealed that the distinct transcriptional signatures demonstrated in the CIC subpopulations were significantly correlated with published stem-cell and epithelial-mesenchymal transition signatures.

**Conclusions:**

The combined results confirm the sensitivity and flexibility of the RNA-Amp™ method and demonstrate the suitability of the approach for identifying clinically relevant signatures in rare, biologically important cell populations.

**Electronic supplementary material:**

The online version of this article (doi:10.1186/1471-2164-15-1129) contains supplementary material, which is available to authorized users.

## Background

Accurate mRNA profiling of single cells can provide a powerful means of broadening our understanding of fundamental biological processes such as cancer and development. A number of recent studies have shown that transcriptional profiling of single cells is possible [1, 2], with three amplification strategies often used: *in vitro* transcription, PCR-based amplification and rolling circle amplification [3–6]. These approaches have been shown to sensitively reflect the biological status of the target cells [7] with for example, analysis of single cells from mouse blastomeres identifying expression of many more genes than previous studies based on hundreds of blastomeres [1]. To take full advantage of recent dramatic technological advances in molecular methods it is essential that these single cell profiling approaches are truly representative of the initial cell amplified, and are also compatible with a broad range of downstream analytical readouts. However, the reproducibility and cross-platform performance of the material generated from these approaches has not generally been confirmed, often because of the limited amounts of material generated. Early single cell studies utilized cDNA microarrays [8] which enable quantification of tens of thousands of known genes [9, 10]. However, this technology has limitations including a restricted fold-range of detection and potential cross-hybridisation between similar sequences [11], as well as being restricted to the probe sets present on the array. The utilization of next generation sequencing (NGS) approaches has the capability of identifying all expressed sequences, achieving massive dynamic ranges, having resolution down to the single nucleotide level [11–13], and has been adapted for single cell transcription studies [1–3]. A third platform that has been used to analyse transcriptional signatures of single cells is high-density qPCR, which provides a more restricted but targeted approach with a wide dynamic range and can be readily transferred to a clinical setting [14]. Each of these approaches has strengths and weaknesses, but the potential to address different questions with regards to single cell analysis.

The ability to transcriptionally profile single cells is of particular value for studying rare, but clinically important cells such as circulating tumour cells (CTC), which can be present at levels as low as ≥1 cell per milliliter of peripheral blood (reviewed in [15]) and cancer initiating cells (CIC), which can comprise less than 1% of the total tumour [16, 17]. Single cell RNA profiling of CTCs and CICs has the potential to provide a means to dissect tumor heterogeneity and identify pathways and genes associated with “stemness” and properties linked to metastasis development and treatment resistance [18–20].

To enable us to accurately and sensitively profile these rare cells we initially compared three commercially available RNA-Amplification protocols to determine the most sensitive and reproducible approach when amplifying single cell equivalent amounts of RNA (25-50 pg). These experiments showed the EpiStem RNA-Amp™ kit to be the most robust. We then further tested this protocol by comparing data generated from MCF7 and MCF10A single cell amplified products on Affymetrix arrays, high density qPCR and NGS (RNA-Seq) to unamplified controls to evaluate its utility across a range of relevant technology platforms. Reproducible transcriptional profiling was seen across all platforms. Having demonstrated the accuracy and reproducibility of the approach we further demonstrated its potential clinical utility through the analysis of highly enriched CICs, sorted from a NSCLC patient-derived xenograft (NSCLC-PDX), according to different surface markers to dissect heterogeneity within the CIC pool (and possibly identify properties of metastatic CICs). RNA-Seq analysis of NSCLC-PDX CICs at the level of 10 cell input revealed clear CIC specific expression patterns with a strong link to previously documented stem cell and epithelial to mesenchymal transition (EMT) profiles [21, 22], confirming the clinical usefulness of the methodology.

## Results

### Comparison of three RNA-Amplification protocols at the single cell level

**Figure 1:**
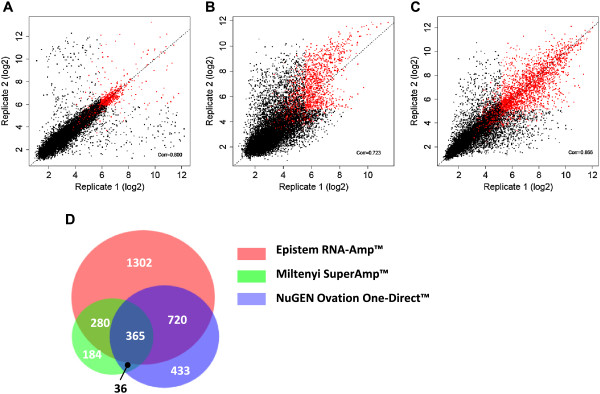
**Transcriptional profiling of RNA-Amplified MCF7 RNA using three different protocols.** Replicate samples of 25-50 pg MCF7 RNA were RNA-Amplified using three commercial kits and 5 μg of the resulting cDNA was analysed on Affymetrix arrays. **(A)** Miltenyi SuperAmp™ replicates showed 865 genes present across both samples with a correlation of 0.800, **(B)** NuGEN Ovation One-Direct™ identified 1554 with a correlation of 0.723 and **(C)** EpiStem RNA-Amp™ identified 2667 present with a correlation of 0.866. **(D)** Venn diagram showing overlap of genes present in both replicates of Miltenyi, NuGEN and EpiStem samples (all analysis based on p ≤ 0.05).

As the aim of our study was to identify a flexible, sensitive and reproducible protocol that could be used to transcriptionally profile at the single cell level initial experiments aimed to directly compare cDNA generated using three kits that were commercially available and had been described for use at the single cell level (Miltenyi μMACS™ SuperAmp™, NuGEN Ovation® One-Direct System and EpiStem RNA-Amp™). To this end, single cell equivalent amounts (25-50 pg) of pooled RNA isolated from the human epithelial cell lines MCF7 were amplified in duplicate and 5 μg of cDNA from each sample run on an Affymetrix array. Bioinformatic analysis of Miltenyi SuperAmp™ material identified 865 expressed genes present in the duplicate samples with a correlation of 0.8 between the samples (Figure 1A), NuGEN Ovation® One-Direct identified 1554 expressed genes with a correlation of 0.723 (Figure 1B) and EpiStem RNA-Amp™ identified 2667 expressed genes with a correlation of 0.866 (Figure 1C). Comparison of the genes identified by each protocol showed 74.6% (645 of 865) of the expressed genes seen in the Miltenyi SuperAmp™ samples and 69.9% (1085 of 1554) found in the NuGEN Ovation® One-Direct samples were also identified in the EpiStem RNA-Amp™ samples, with a total of 67.6% (1365 of 2018 genes) of all genes identified by either SuperAmp™ and/or Ovation® One-Direct being picked up in the RNA-Amp™ material (Figure 1D). Since these data indicated the EpiStem RNA-Amp™ system gave the most sensitive and reproducible results it was chosen for further evaluation.

### Generation of high yields of reproducible cDNA from single cells

**Figure 2:**
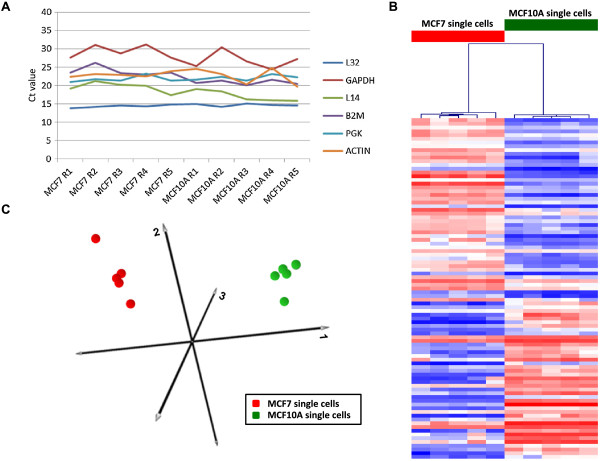
**Transcriptional profiling of RNA-Amplified MCF7 and MCF10A single cells. (A)** Real-time PCR of RNA-Amp™ samples showing sensitive and consistent detection of 6 “housekeeping genes” in all single cell samples. **(B)** A heat map presentation of differentially expressed genes (LIMMA FC > 2, FDR < 0.01) detected in the Affymetrix array data from the group of 10 single cell MCF7 and MCF10A samples with blue indicating the lowest detected, red indicating the highest detected and white the midpoint. **(C)** PCA plot generated from the entire single cell Affymetrix array data set showing separation of all MCF7 and MCF10A samples.

To further determine the sensitivity of the RNA-Amp™ kit we tested the protocol on single cells from two human epithelial cell lines MCF7 and MCF10A (5 single cells for each cell line). All samples were subjected to RNA-Amp™ and the resulting cDNA analysed by real-time PCR for the expression of 6 house keeper genes (Figure 2A, Additional file 1: Table S1). This showed consistent amplification of all amplicons, down to single cell input for all replicates, indicating reliable cDNA products were obtained that were suitable for further analysis.

### Generation of single cell Affymetrix array data

RNA-Amp™ resulted in average yields of 4.1 μg of cDNA from single cell inputs (range 3.0-5.1 μg). The large amount of material generated enabled the use of Affymetrix arrays to determine the reproducibility of the RNA-Amp™ method. Bioinformatic analysis of Affymetrix expression data from 5 single MCF7 and 5 single MCF10A cells identified 92 genes differentially expressed between the two cell lines (LIMMA, FC > 2, FDR < 0.01), 50 showing higher expression in MCF7 and 42 showing higher expression in MCF10A (Figure 2B), with PCA analysis of the entire data set clearly separating all samples according to cell line (Figure 2C), highlighting good reproducibility across all 5 single cell inputs from each cell line.

### Comparison of single cell results to reference data

Having demonstrated the practical utility of RNA-Amp™ using single cell inputs we next set out to further determine its characteristics by comparing the transcriptional profiles generated from RNA-Amp™ single cell samples to RNA-Amp™ cDNA from purified RNA equivalent to approximately 100 cells equivalent and unamplified reference RNA. For the unamplified reference samples we utilised a previously published data set generated from Affymetrix arrays of 10 μg of unamplified RNA from each cell line [23]. For the 100 cell equivalent input we performed RNA-Amp™ on 1 ng of purified RNA from each cell line (5 replicates for each cell line), which was then analysed on Affymetrix arrays as described above.

**Figure 3:**
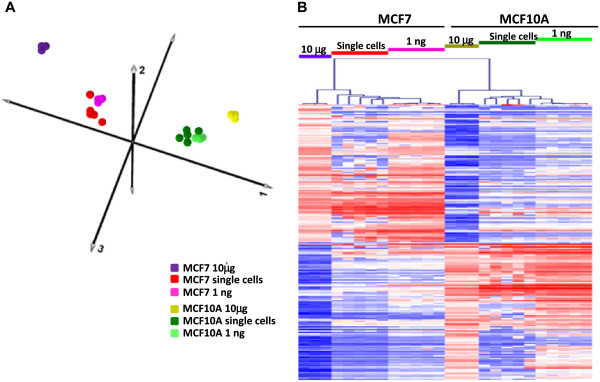
**Comparison of differential expression between amplified and unamplified samples. (A)** PCA analysis of DE genes (LIMMA FC > 2, FDR <0.01) identified from Affymetrix array analysis of 10 μg MCF7 and MCF10A RNA samples aligned with the corresponding Affymetrix array data for the single cell and 1 ng amplified MCF7 and MCF10A samples showing clear clustering according to cell type. **(B)** Heat map of hierarchical clustering of the top 200 differentially expressed genes identified from Affymetrix array analysis of 10 μg MCF7 and MCF10A RNA samples aligned with the corresponding Affymetrix array data for the single cell and 1 ng amplified MCF7 and MCF10A samples. Heat map colour scheme as described in Figure 2B.

From these Affymetrix array data sets we first selected all of the significantly differentially expressed genes identified in the 10 μg reference data (2202 genes, LIMMA FC > 2, FDR <0.01) and aligned these with the corresponding Affymetrix array data for the single cell and 1 ng amplified MCF7 and MCF10A samples and performed PCA analysis. This analysis showed strong grouping of the two cell lines irrespective of input material (Figure 3A). We next identified the 200 highest differentially expressed genes (LIMMA, FC > 2, FDR < 0.01) in the reference data set (100 MCF7 > MCF10A, 100 MCF10A > MCF7) and compared the expression profiles of these transcripts to our single cell and 1 ng data sets (Figure 3B). Hierarchical clustering based on the top 200 differentially expressed genes from the 10 μg reference data again showed clear separation of the two cell lines for all template inputs, with strong correlation seen between the 10 μg reference differentially expressed data and the 1 ng data (Pearson correlation 0.89), and between the 10 μg reference and the single cell data (Pearson correlation 0.78).

### Generation of RNA-Seq data from single cells

To determine the suitability of RNA-Amp™ material for NGS, and to compare NGS data to microarray data from the same samples we subjected cDNA from the 5 MCF7 single cells and 5 MCF10A single cells used for Affymetrix array analysis to SOLiD RNA-Seq NGS. All samples produced high quality NGS data with an average coverage of 20 × 10^6^ uniquely mapped reads per sample (range 17-22 × 10^6^, Additional file 1: Table S2). Bioinformatic analysis of the data identified 650 genes showing elevated expression in MCF7 single cells and 794 showing elevated expression in MCF10A single cells (EdgeR, FC > 2, FDR < 0.05, Additional file 1: Table S3).

**Figure 4:**
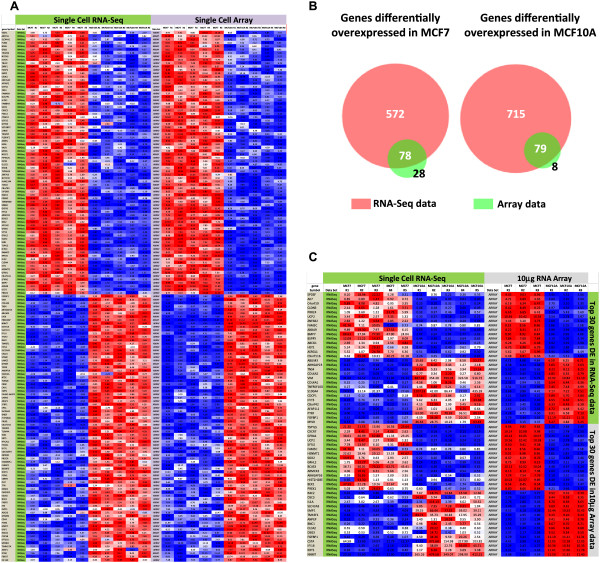
**Comparison of RNA-Seq and Microarray data from single cells. (A)** A comparison of RNA-Seq and Affymetrix array data generated from the same amplified single cell samples. The overall correlation (Pearson) of the MCF7/MCF10A ratio between RNA-Seq and Affymetrix array data sets for the 157 genes examined was 0.95. **(B)** Venn diagrams showing overlaps of differentially expressed genes identified by RNA-Seq and Affymetrix array analysis (FC > 2, FDR < 0.05 for both data sets) highlighting the larger number of DE genes identified in the RNA-Seq data set. **(C)** A comparison of single cell RNA-Seq data and10 μg RNA Affymetrix array data showing the expression profiles of the top 30 differentially expressed genes identified by RNA-Seq or 10 μg RNA Affymetrix array data (all data FC > 2, FDR threshold 0.05). Heat map colour scheme for **(A)** and **(C)** as described in Figure 2B.

To assess cross-platform performance of the amplified material we then compared the expression data generated from the single cell RNA-Amp™ cDNA samples from both the RNA-Seq and the Affymetrix array data (all data analysed FC > 2, FDR < 0.05). This analysis identified 157 genes showing significant differential expression in both data sets (Additional file 1: Table S4). Comparison of these 157 genes showed consistent patterns of differential expression across both platforms, with an overall Pearson correlation between the RNA-Seq and Array data of 0.92 (Figure 4A). From this analysis more differentially expressed genes were identified from the RNA-Seq data set than the Affymetrix array data, with the majority of genes identified in the array data set also identified in the RNA-Seq data (Figure 4B).

To determine whether the additional differentially expressed genes identified by RNA-Seq were true transcriptional differences and not technical error, we compared the RNA-Seq data from the single cells to the 10 μg reference microarray data set. All significant differentially expressed genes were identified in both data sets using LIMMA (Array) and EdgeR (RNA-Seq) with a FC > 2 and a FDR < 0.05. A combined total of 597 differentially expressed genes were identified, with an overall Pearson correlation for differential expression (average MCF7 divided by the average of MCF10A samples) between the data sets of 0.89. To directly compare patterns of expression we identified the 30 most differentially expressed genes in the single cell RNA-Seq data, and then compared their expression in the 10 μg reference data. We then performed the complementary analysis by identifying the 30 most differentially expressed genes in the 10 μg reference data and compared their expression in the in the single cell RNA-Seq data (Figure 4C). A highly significant correlation (Pearson 0.90) was identified between all 60 genes, with similar expression patterns seen in the single cell RNA-Seq and the 10 μg reference data (Figure 4C).

### High density qPCR of single cell RNA-Amp™ samples

Having shown that RNA-Amp™ material was amenable to both array and NGS approaches, and produced sensitive, reproducible and accurate results, we finally wanted to investigate its utility for focussed high density qPCR approaches. To this end, we identified a panel of 173 qPCR amplicons (Additional file 1: Table S5) based on the single cell microarray data set and examined expression using the WaferGen SMARTChip high density qPCR platform. In addition to the single cell RNA-Amp™ samples we also included three unamplified 2 μg reference samples and three 1 ng RNA-Amp™ samples.

**Figure 5:**
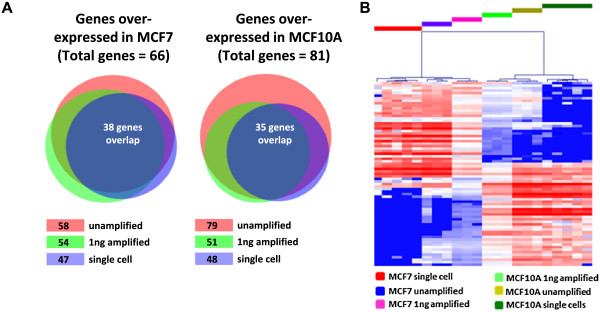
**High-density real-time PCR analysis of differentially expressed gene signatures. (A)** Venn diagrams showing the overlap of differentially expressed MCF7/MCF10A genes detected by high density qPCR of: unamplified cDNA from 1 μg RNA; RNA-Amp™ cDNA from 1 ng RNA; and RNA-Amp™ cDNA generated from single cells. Numbers in boxes represent the number of genes upregulated in that template type. **(B)** Bioinformatic analysis of the real-time PCR data identified 73 genes differentially expressed between MCF7 and MCF10A across all template types (LIMMA FC > 2, FDR < 0.01) and hierarchical clustering clearly separated the two cell lines. Heat map colour scheme for **(B)** as described in Figure 2B.

LIMMA analysis of the WaferGen qPCR data identified 66 genes upregulated in the MCF7 population, and 81 upregulated in the MCF10A population (FC > 2, FDR < 0.01). When we looked at the overlap between the different template types we found that 38 (58%) of the MCF7 > MCF10A genes were determined to be significantly upregulated for all sample types and 35 (43%) of the MCF10A > MCF7 gene set were also seen to be significantly changed in all sample types (Figure 5A). Hierarchical clustering of the combined data set clearly separated the two cell lines for all template inputs, including single cells and reference RNA samples (Figure 5B).

### Profiling of highly enriched lung cancer stem cells

**Figure 6:**
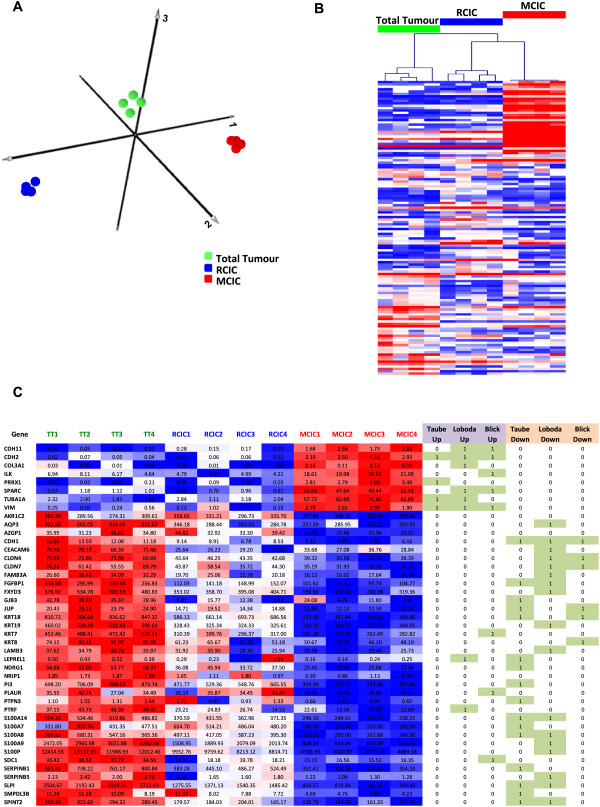
**Transcriptional profiling of fractionated NSCLC-PDX subpopulations. (A)** PCA analysis of total RNA-Seq data from NSCLC-PDX fractionated samples showed clear separation of the metastasis associated cancer initiating cells (MCIC) and resident cancer initiating cells (RCIC) from unfractionated total tumour (TT). **(B)** Heat map of hierarchical clustering of top differentially expressed genes (50 TT v RCIC, TT v MCIC, RCIC v MCIC, EdgeR FC > 2, FDR < 0.05) illustrates clear separation of the three populations and a set of genes with the most striking change seen between TT and MCIC samples. Heat map colour scheme for **(B)** as described in Figure 2B. **(C)** Summary of EMT signature genes found to be differentially expressed (FC > 2, p < 0.05) in NSCLC-PDX fractionated samples with correlation between differentially expressed genes identified in MCIC and three published EMT signatures highlighted (boxed green). Column headings are: MCIC - metastasis associated cancer initiating cells; RCIC- resident cancer initiating cells; TT - unfractionated total tumour (TT); Taube *et al.* - EMT genes identified by Taube and colleagues [27]; Loboda *et al.* – EMT genes identified by Loboda and colleagues [21]; Blick *et al.* - EMT genes identified by Blick and colleagues [22].

Having established the sensitivity and accuracy of the RNA-Amplification approach on cell lines, we sought to test its utility with clinically relevant samples. For this we set out to identify genes associated with biologically distinct cell subpopulations obtained from patient-derived non-small cell lung cancer (NSCLC) xenografts (NSCLC-PDX). We have recently demonstrated that within CD133+ CICs from NSCLC-PDXs, we can define a fraction of CD133+/EpCAM+ cells that represent the resident cancer initiating cells or RCIC subpopulation [19], as well as a population of metastatic-associated cancer initiating cells (MCIC) with surface markers CD133+/CXCR4+/EpCAM- which show increased potential for metastasis formation ([20] and data in progress). To increase our understanding of the molecular make-up of these populations we dissociated a NSCLC-PDX tumour and subjected it to flow cytometric fractionation to obtain unfractionated total tumour (TT), as well as RCIC and MCIC enriched fractions (1% and 0.02% of total tumour cells respectively). All cell samples were sorted directly into Complete Lysis solution (CLS – Materials and Methods) and stored at −80°C. Following thawing, lysate volumes equivalent to 10 cells were subjected to RNA-Amp™ and RNA-Seq analysis of the resulting cDNA was carried out. From the RNA-Seq data PCA analysis and hierarchical clustering (Figure 6A and 6B) of the protein coding genes showed clear separation of all samples, with 1,126 genes identified which showed statistically significant differences in expression (EdgeR, FC > 2, FDR < 0.05) between the TT, RCIC and MCIC samples (Additional file 1: Table S6). From Figure 6B it can be seen that there are clear differences between each group and that the most conspicuous differences are seen between the total tumour and the MCIC samples.

To determine the biological relevance of these differentially expressed gene sets we next asked whether genes showing increased (MCIC UP) or reduced (MCIC DOWN) expression in the MCIC population, compared to the TT samples, are enriched for specific pathways, or overlap with published RNA profiles by submitting the top 100 differentially expressed genes for DAVID [24] and GeneSigDB [25] analysis. DAVID pathway analysis showed that MCIC UP genes were linked to cytoskeleton, ribosomal processing, glutathione transferase and, to a lesser extent, RNA splicing and tubulin (Additional file 1: Table S7). This data includes two genes of interest, GSTP1 and BRCA1, that were highly expressed in MCIC cells (mean RPKM 997 and 0.84 respectively) compared to the TT cells (mean RPKM = 8.1 and 0.08 respectively. Whereas MCIC DOWN genes only showed a weak link to mitochondria (Additional file 1: Table S8). From the GeneSigDB analysis both MCIC UP and MCIC DOWN genes showed highly significant matches to stem cell and EMT profiles (Additional file 1: Tables S9 and S10). The EMT signatures included a signature seen in CD44(hi)/CD24(lo/-) enriched breast cancer stem cells [16] and an EMT core signature produced by overexpression of Twist, Snail, Gsc and TGF-β1 [26]. The overlap between the differentially expressed genes identified in the RNA-Seq data set and other published studies is shown in Figure 6C. This figure summarises genes showing a statistical change in the MCIC samples which also correspond to published EMT signature genes [21, 22, 27].

## Discussion

Single cell whole transcriptome profiling approaches have been in place for over two decades [5, 28, 29] and have led to the identification of novel genes and greater insight into cellular processes ([30, 31], reviewed [32]). More recently, single cell transcriptome and genomic approaches have been combined [4] and single cell RNA-Seq approaches have been developed [1–3]. Despite the demonstrable success of single cell analysis the technical requirements needed for representative amplification of single cells and the downstream analysis remain a considerable barrier for widespread implementation in the research community. Here we have compared three commercially available RNA-amplification kits and identified a simple and flexible single cell mRNA profiling kit (EpiStem RNA-Amp™), which provides microgram amounts of amplified cDNA suitable for analysis using a wide range of downstream platforms, including high density qPCR arrays, Affymetrix arrays and RNA-Seq. We have used this approach to successfully generate representative cDNA from single cells and single cell equivalents (25-50 pg), 1 ng purified RNA (equivalent to ~100 cells) as well as from 10 cell pools of directly fractionated tumour cells with comparable transcriptional profiles seen across all platforms and all template inputs.

Initial experiments compared gene expression profiles generated from single cell equivalent amounts of pooled MCF7 RNA using three commercially available kits. The use of pooled RNA reduced the level of biological variation that could be typically expected at the single cell level, meaning differences between the duplicate samples could be mainly attributed to technical variation. Direct comparison of the three kits found that the EpiStem RNA-Amp™ kit was most sensitive, identifying 2667 expressed genes compared to 1554 and 865 with the NuGEN and Miltenyi generated cDNA respectively. It was also the most reproducible with the correlation between duplicate samples being 0.866, compared to 0.723 and 0.8 for the NuGEN and Miltenyi samples. The data from these initial experiments lead us to focus on the EpiStem RNA-Amp™ kit for further, detailed evaluation.

Having found the EpiStem RNA-Amp kit to be sensitive and reproducible we then went on to determine how representative the amplified material was of the initial transcriptome of the cell. From Affymetrix array analysis we established that genes identified using conventional profiling starting with 10 μg of purified RNA (equivalent to ~10^6^ cells [23]) showed a similar pattern in amplified products from either single cells or 1 ng RNA (Figure 3), confirming that the protocol is ‘fit for purpose’ and will identify RNA changes reflecting the biological status of the starting sample. This was an important result as it confirmed that the data generated from the amplified material was biologically representative. The next question addressed was whether the amplified material could be analysed across a range of different platforms and whether the different platforms gave comparable transcriptional profiles. A comparison of genes identified by Affymetrix arrays or RNA-Seq analysis of the same single cell amplified cDNA revealed similar patterns of expression (Figure 4A) demonstrating platform independence. Interestingly, substantially more differentially expressed genes were identified by RNA-Seq analysis than with the Affymetrix microarrays (Figure 4B). The reason for the increased numbers of genes detected by RNA-Seq is not completely clear, but likely reflects the increased sensitivity and lower background of the method as well as the lack of 3’ bias and matches as seen in previously published studies [1]. The additional differentially expressed genes identified in the RNA-Seq analysis were shown to be true biological variation between the cell lines by comparing the single cell RNA-Seq data to the 10 μg reference data (Figure 4C), with differentially expressed genes identified within these data sets showing significant correlation (Pearson correlation 0.89). If the additional genes identified by RNA-Seq were due to ‘technical noise’ we would not have expected to have seen enrichment of these transcripts in the reference data set.

Finally we tested the cDNA on a high density qPCR platform, using the WaferGen SMARTChip system that enables analysis of over 5000 wells per run, based on 100 nl reactions. The appeal of qPCR over other platforms is that it has already been established in various clinical settings, including the monitoring of CTCs [33, 34]. In this study we utilised transcriptional profiles identified within our microarray data to design a panel of 173 qPCR amplicons predicted to be differentially expressed between the two target cell lines. We tested the expression signatures in cDNA amplified from single cells, 1 ng of RNA and unamplified RNA and found good concordance across template inputs (Figure 5). These results demonstrate a potential pipeline by which rare, clinically important cells can be transcriptionally profiled using microarrays and/or RNA-Seq analysis of RNA-Amplified material, and targeted expression profiles generated from these data then monitored in multiplexed, high density qPCR assays for clinical utility.

Having proven the robustness and accuracy of the RNA-Amp™ approach we then validated its potential clinical/research utility through analysis of highly fractionated cancer initiating cells from NSCLC-PDX samples. Within primary tumors, CICs are functionally defined as the cellular subset responsible for generation and maintenance of tumours [16], and in most solid tumors represent only a fraction of the total cellular population. Recently it has been proposed that the activation of the epithelial to mesenchymal transition provides tumor cells with stem-like features and dissemination ability, traits needed to carry out the metastatic process [35, 36]. These findings are supported by clinical evidence in breast cancer patients showing that CTCs and bone marrow disseminated tumor cells exhibit EMT and stemness features [37, 38]. Additional analysis of CTCs from breast cancer patients provided evidence for the existence of a definite subset of MCICs, showing stemness and mesenchymal traits that possess the potential to initiate metastases [39, 40]. Previously we have shown that lung cancer contains a population of CD133^+^ cancer initiating cells, within which is a subpopulation of CD133^+^CXCR4^+^ EpCAM^−^ cells that show an increased metastasis formation capability and could represent these clinically important lung MCICs (manuscript in preparation). Therefore, it would be of great interest to determine the transcriptional profile of these rare cells and compare to other subpopulations within the tumour. However, following flow cytometric fractionation typically fewer than 100 MCIC cells are isolated from each tumour, therefore we utilized the Epistem RNA-Amp protocol to generate enough material from these rare cells for transcriptional profiling.

For this we amplified RNA from equivalent to 10 cells input, and following RNA-Seq analysis, which in our validation experiments was found to generate the most data, we were able to clearly distinguish RNA profiles from CICs with an increased potential for metastasis formation (MCIC) from resident CICs and total tumour profiles (Figure 6). Pathway analysis of MCIC differentially expressed genes revealed a clear enrichment for a number of pathways (Additional file 1: Tables S7 and S8), including a strong link to glutathione metabolism. This has potentially important clinical implications since glutathione S-transferase pi 1 (GSTP1) is known to regulate sensitivity to cytotoxic agents (reviewed [41]) and is a significant risk factor for clinical chemotherapy resistance in NSCLC [42]. Since GSTP1 is low in the TT samples and over 100× higher in the MCIC samples (Additional file 1: Tables S6) this may imply selective resistance of MICICs. A comparison of MCIC differentially expressed genes to published RNA expression signatures [35] showed a strong link to known EMT and stem cell profiles (Additional file 1: Tables S9 and S10). MCIC differentially expressed genes overlapped with a range of EMT signatures, including a signature derived from human lung cancer cell lines and shown to be linked with a prognostic signature in colorectal cancer [21], a core signature that is produced by overexpression of Twist, Snail, Gsc and TGF-β1 [36] as well as an EMT signature detected in CD44(hi)/CD24(lo/-) breast cancer stem cells [22] (Figure 6C).

In addition to confirming the reported convergence of EMT signatures and stem cell enrichment in the MCIC population, we also detect stem cell related changes which are not obviously linked to EMT, including increased expression of BRCA1 in both RCIC and MCIC samples (Additional file 1: Table S6). As well as its role in DNA repair and breast cancer susceptibility, BRCA1 has also been implicated in mammary stem-cell self-renewal (reviewed [43, 44]), with deletion of BRCA1 during epidermal development showing that it is required for the development of adult hair follicle stem cells [45]. Thus, these findings support the existence of different subsets of lung CICs that can be distinguished from total tumor cells by a common stemness signature, whereas the co-expression of the mesenchymal signature was able to define those tumor stem cells endowed with the greatest dissemination and metastatic potential. These data strongly suggest that by utilizing the RNA-Amp™ protocol to amplify RNA from these rare cells has enabled us to accurately determine their transcriptional signatures and further reveal the genes and pathways involved in tumourigenesis.

## Conclusions

We have shown through detailed transcriptional profiling of single cells from two control cell lines, across multiple platforms, the value and robustness of the profiling approach we describe. These results confirm that RNA amplification from single cells is readily achieved, the material generated accurately reflects the transcriptional status of the initial cell, can be used for RNA-Seq and microarray analysis and that these data sets can be used to generate targeted expression panels that are amenable to real-time PCR analysis. This process mirrors the pipeline we envisage could be used to optimally transfer clinically important findings into a therapeutic setting.

In addition, we have validated the approach through the characterization of potential metastatic cancer initiating cells isolated from a NSCLC-PDX model, with this analysis identifying a panel of EMT and stem-cell associated genes with potential roles in metastatic spread. This highlights the utility of the protocol in better understanding the biology of clinically important, rare cellular populations.

## Methods

### Cell culture and FCM sorting of single MCF7 and MCF10A cells

The human epithelial cell lines MCF7 and MCF10A were grown in DMEM (Gibco, Paisley, Scotland) supplemented with 10% heat-inactivated FCS (5% Horse serum for MCF10A cells), 2.5% HEPES buffer (pH 7.2), 0.1% β-mercaptoethanol and 2 mM L-glutamine (all from Sigma, UK). For MCF10A cells, Insulin (10 μg/ml), EGF (20 ng/ml), Cholera Toxin (100 ng/ml) and Hydrocortisone (500 ng/ml) were also added (all from Sigma, UK). Both cell lines were maintained in a 5% CO_2_ humidified incubator at 37°C and were routinely tested for the presence of mycoplasma. For single cell experiments each cell line was FACS sorted on a BD Influx Sorter (BD, California, USA) machine directly into 96-well plates containing 5 μl of Complete Lysis Solution (CLS) (10 mM Tris, 1 mM EDTA, 5% Igepal-CA (v/v), Roche Protector RNAse inhibitor cocktail).

### Representative cDNA amplification

#### EpiStem RNA-Amp™

RNA (1 ng-25 pg) or cell lysates in CLS were adjusted to 6.75 μl volume and RNA-Amplified using the EpiStem RNA-Amp™ Kit according to the manufacturers protocols (EpiStem, Manchester, UK). Briefly the samples underwent oligo-dT priming and 5’ capping prior to ×35 cycles of PCR amplification using the conditions 90°C 30 sec, 42°C 2 min and 72°C 6 min. Following amplification, all samples were purified using a MoBio UltraClean® PCR Clean-Up Kit (Carlsbad, CA, USA) and quantified on a NanoDrop spectrophotometer.

### Miltenyi μMACS™ SuperAmp™

1 μl MCF7 RNA (25 pg μl^−1^) was added to 5.4 μl of freshly prepared Incubation Buffer and RNA-Amplified using the Miltenyi μMACS™ SuperAmp™ according to the manufacturers protocols (Miltenyi, Gladbach, Germany). Briefly, the samples underwent in-column cDNA synthesis and purification, cDNA tailing and finally cDNA amplification using the conditions 78°C 30 sec followed by ×20 cycles of 94°C 15 sec, 65°C 30 sec, 68°C 2 min, then ×21 cycles of 94°C 15 sec, 65°C 30 sec, 68°C 2 30 sec + 10 sec/cycle with a final incubation of 68°C for 7 min. Following amplification, cDNA was purified using a Roche High Pure® PCR purification Kit (Basel, CH) and quantified on a NanoDrop spectrophotometer.

### NuGEN ovation® one-direct system™

5 μl MCF7 RNA (10 pg μl^−1^) was added to 2 μl of First Strand Primer mix and RNA-Amplified using the NuGEN Ovation® One-Direct System™ Kit according to the manufacturers protocols (NuGEN, CA, USA). Briefly, the samples underwent first strand cDNA synthesis, second strand cDNA synthesis, SPIA Amplification followed by Post-SPIA modification using the conditions 4°C 1 min, 30°C 10 min, 42°C 60 min then 75°C for 10 min. Following amplification, cDNA was purified using a QIAGEN Minelute column (Hilden, Germany) and quantified on a NanoDrop spectrophotometer.

### Real-time PCR analysis of house keeper transcripts

The expression levels of 6 house keeper gene transcripts was analysed using real-time PCR. Briefly, following RNA-Amp™ amplification each sample was diluted 1:100 and 1 μl of the resulting sample used as the template in a real-time PCR reaction. All reactions contained 350nM forward and reverse primer and 1x SYBR Green Master mix (Applied Biosystems, Warrington, UK), run on an ABI 7900 and data analysed using SDS2.4 software (Applied Biosystems, Warrington, UK). The primers used are shown in Table S1.

### Affymetrix DNA microarrays

Following amplification the cDNA was fragmented and Biotin-labeled using the EpiStem RNA-Amp™ labeling kit (EpiStem, Manchester, UK) and the hybridisation cocktail containing 5 μg of biotin cDNA heated to 99°C for 5 mins. The hybridisation cocktail was then transferred to 45°C for 5 mins and centrifuged at maximum speed for 5 mins to remove insoluble material. Samples were then hybridised to HU133plus2 arrays for 16 hours and then stained with SAPE using a biotin targeted antibody step and washed according to the EukGE_Ws2v4_450 fluidics protocol from Affymetrix. Samples were then scanned in an Affymetrix 3000 scanner.

### RNA-Seq NGS

To ensure all cDNA generated using the Epistem RNA Amp kit was double stranded, one cycle of re-amplification was performed (95°C for 2 mins, 55°C for 1 mins followed by 72°C for 15 mins). A library was then prepared using 1ug of the dsDNA in the Life Technologies 5500 SOLiD Fragment Library Core kit (Life Tech, Paisley, UK) according to the manufacturer’s instructions. The libraries were quantified using the Life Technologies SOLiD Library Taqman Quantitation kit and Emulsion PCR was performed using the Life Technologies SOLiD EZ Bead System (Life Tech, Paisley, UK). 50 bp single read sequencing was carried out on the Life Technologies 5500XL SOLiD System (Life Tech, Paisley, UK).

### High density qPCR

Gene expression was assessed using the SmartChip Real-Time PCR System (WaferGen BioSystems, Fremont, USA). Sample and assay mixes were prepared with SensiFAST™ SYBR Hi-ROX (Bioline, London, UK) in 384-well source plates using a Freedom Evo 150 robot (Tecan, Mannedorf, Switzerland). Assay and sample mixes were then automatically loaded into the nanowells of a MyDesign SmartChip with WaferGen’s MultiSample NanoDispenser using a ‘384 assays × 12 samples’ dispensing layout. The final reaction volume per nanowell was 100 nl, with an equivalent of 100 pg unamplified cDNA (total RNA equivalents) loaded per reaction. SmartChips were run in the SmartChip Cycler, and the cycling conditions were comprised of 3 minutes activation at 95°C, and 40 cycles of 30 seconds at 95°C and 60s at 60°C, followed by a dissociation curve analysis from 60°C to 95°C. Cq values, generated by the software from the SmartChip Cycler were used for down-stream data-analysis.

### Isolation of NSCLC-PDX CIC populations

Patient derived xenografts (PDX) were established from lung cancer primary tumors and expanded as previously described [46]. Briefly, after approval from the Internal Review and the Ethics Boards of the Fondazione IRCCS Istituto Nazionale Tumori, samples of primary NSCLC were obtained from patients undergoing surgical resection following receipt of informed consent in compliance with the Declaration of Helsinki. Each sample was cut in small pieces (25–30 mm^3^) and implanted subcutaneously using a trocar gauge in the flank of female SCID mice. PDXs were then expanded in vivo through successive rounds of transplantation from donor to recipient mice. To obtain single cell suspension, PDXs were mechanically and then enzymatically digested in a solution of collagenase IV (5 mg/ml) and DNAse (100U/ml) (Sigma-Aldrich) in DMEM/F12 (Lonza) for 1 h at 37°C. Partially digested tissue was filtered through a 100 μm cell strainer (BD Falcon) and red blood cells were removed by Lysing Buffer 1X (BD Bioscience).

Single cell suspensions from dissociated NSCLC-PDXs were washed and incubated in staining solution 1% BSA and 2 mM EDTA with specific antibodies at appropriate dilutions for 30 min at 4°C: PE anti-human CD133/1, FITC anti-human CD326 (EpCAM) (Miltenyi Biotech), APC anti-human CD187 (CXCR4) (BD Pharmingen), Alexa Fluor® 488 anti-human HLA-ABC (BD Pharmingen), PerCP-eFluor 710 anti-mouse MHC class I (e-Bioscience). Prior to sorting, cells were resuspended to a final concentration of 10 ×10^6^ cells/ml in Hepes Buffered Saline (HBS) (Lonza) + 0.1% B27 Supplements (Gibco) and 7-AAD viability staining solution (1:10) (e-Bioscience) for dead cells exclusion.

PDX-cells were sorted with FACSAria (Becton Dickinson) into a chilled 96-well plate (Corning Incorporated). For sorting of different CIC fractions, an initial gate excluding doublets, dead cells and mouse MHC class I^+^ cells was set. Then, within the gate of human viable CD133^+^ cells, the fraction of EpCAM^+^ and CXCR4^+^EpCAM^−^ cells were identified and sorted. Total tumor population was isolated based on human HLA-ABC^+^ expression. For each cell fraction, 300 cells were directly sorted in 30 μl of CLS per well and within 1 h approximately 100 cells/10 μl were transferred into 0.5 ml tubes and stored to −80°C.

### Bioinformatics analysis of microarray data

MCF7 cDNA was generated by three different protocols, NuGEN, EpiStem and Miltenyi and each sample had two replicates. The probe-level expression values for each replicate of one sample were obtained from the Affymetrix HG133plus2 array and were normalised and summarised as the expression values at the probeset level using the RMA method [47], implemented in the Affymetrix’s tool ‘apt-probeset-summarize’. The Bioconductor [25] package panp was used to determine that a probeset was present in one replicate if the p-value calculated by panp was less than 0.05. For each replicate, the present probesets were mapped to the genes using the ENSEMBL human gene annotation database version 70 via the BioConductor package annmap [48]. Gene lists for one sample were compiled from the two replicates, containing the genes which were present in both replicates.

Data for the MCF7 and MCF10A samples were generated using Affymetrix Human HGU133plus2 arrays (referred to as Affymetrix arrays throughout). Data were normalised and summarised using RMA [47], as implemented by Affymetrix Power Tools (APT) software package (http://www.affymetrix.com/estore/partners_programs/programs/developer/tools/powertools.affx) using the ‘apt-probeset-summarize’ default parameter setting. Gene level summaries were computed as the geometric mean of all probesets mapping to a gene, as defined by annmap [48], using ENSEMBL version 70 as the source of underlying genome annotation. The empirical Bayes statistics [49] implemented in the Bioconductor package LIMMA [50] were used to identify protein-coding genes showing differential levels in MCF7 and MCF10A samples (FDR < 0.01; FC > 2).

### Bioinformatics analysis on the single cell RNA-Seq data

50mer single-ended strand specific RNA-Seq data were generated using a SOLiD 5500XL sequencing machine and aligned to human genome hg19 using SHRIMP2 [51, 52]. Reads that aligned to multiple loci were discarded. Subsequent analyses were performed using R and Bioconductor [26]. Between 7.6 and 10.4 million reads aligned uniquely for each sample in the MCF10A and MCF7 dataset. For the single cell lung cancer samples, reads were aligned to the human (hg19) and mouse genomes (mm9) separately. For the 4 TT and 4 RCIC samples, 38% - 48% of reads mapped uniquely to the human genome, while 5.8% - 7.1% mapped uniquely to mouse. For the 4 MCIC samples, 12 - 13% of reads aligned uniquely to the human genome and 37 - 42% of reads to mouse. We therefore discarded reads that aligned both to human and mouse genomes in order to retain, for each of the 12 samples, only those reads that aligned uniquely and exclusively to the human genome. Following this filtering step, the number of reads retained for the 4 MCIC samples ranged from 1.7 - 3.6 million and between 5.2 and 14.2 million reads remained for the 8 TT and RCIC samples.

For each sample, reads were positioned relative to annotated genes from ENSEMBL version 70, using the annmap database and Bioconductor package [48], and the number of reads hitting exonic regions was counted for each gene. These data were then used to identify differentially expressed (DE) genes using the Bioconductor package EdgeR [53] (FDR < 0.05; FC > 2; exact test method [54]). Four gene lists were produced: MCF10A vs. MCF7, TT vs. MCIC, TT vs. RCIC, and MCIC vs. RCIC samples.

#### Accession numbers

The MCF7 and MCF10A single cell and 1 ng Affymetrix microarray, RNA-Seq and WaferGen qPCR data have been deposited to the NCBI under accession number GSE52717.

## Electronic supplementary material

Additional file 1: Table S1: Housekeeper genes primer sequences. **Table S2.** Single cell RNA-Seq mapped reads information. **Table S3.** Single cell RNA-Seq EdgeR data. **Table S4.** Differentially expressed genes identified in both RNA-Seq and Affymetrix Array analysed data of single MCF7 & MCF10A cells. **Table S5.** WaferGen primer sequences for 173 amplicons. **Table S6.** NSCLC-PDX RNA-Seq EdgeR data. **Table S7.** MCIC up-regulated genes DAVID analysis. **Table S8.** MCIC down-regulated genes DAVID analysis. **Table S9.** MCIC up-regulated genes GeneSigDB analysis. **Table S10.** MCIC down-regulated genes GeneSigDB analysis. (XLSX 819 KB)

## Abbreviations

CIC: Cancer initiating cell
CTC: Circulating tumour cell
DE: Differentially expressed
EMT: Epithelial to mesenchymal transition
FCM: Flow cytometry
MCIC: Metastatic associated cancer initiating cell
NGS: Next generation sequencing
NSCLC: Non-small cell lung cancer
NSCLC-PDX: Non-small cell lung cancer patient-derived xenograft
RCIC: Resident cancer initiating cell
TT: Total tumour.
